# ‘You may be a mother, but you’re also a child.’– the importance of unintended pregnancy of underaged women in Germany for sexual and reproductive health: a biographical narrative analysis

**DOI:** 10.1186/s12978-025-02242-4

**Published:** 2026-01-28

**Authors:** Kristina Winter, Martin Nowak, Nele Schneider, Dennis Jepsen, Petra J. Brzank

**Affiliations:** 1 Institute for Social Medicine, Rehabilitation Sciences and Health Services Research, University of Applied Sciences Nordhausen, Nordhausen, Germany; 2 Institute of Medical Sociology, Interdisciplinary Center of Health Sciences, Medical Faculty, Martin-Luther-University Halle-Wittenberg, Halle (Saale), Germany; 3 Hildesheim/Holzminden/Göttingen, Faculty of Management, HAWK University of Applied Sciences and Arts, Social Work, Construction, Holzminden, Germany

**Keywords:** Adolescents, Teenage pregnancy, Reproductive health and rights, Abortion, Domestic violence, Teen dating violence (TDV), Adverse childhood experiences (ACE), Qualitative research, Documentary method

## Abstract

**Background:**

Adolescence is a formative period in which self-concept and sexual identity are developed. Unintended teenage pregnancies represent a sensitive and stigmatized issue, often associated with major psychosocial challenges. The aim of this study is to explore the lived experiences of women with unintended teenage pregnancies in Germany, with a particular focus on adverse childhood experiences (ACE) and Teen Dating Violence (TDV).

**Methods:**

The analysis is based on five biographical narrative interviews conducted within the ELSA project (November 2020–April 2024), which aimed to investigate how unintended pregnancies are managed and to identify needs for counseling and care. Using the documentary method, we reconstructed the meaning of communicative and conjunctive knowledge in adolescents’ narratives and compared these orientations across four comparative dimensions: childhood biography, handling of the unintended pregnancy, construction of intimate relationships, and construction of minority.

**Results:**

Two contrasting orientation types were reconstructed across the dimensions, shaped by how adolescents processed external framings. Type 1 (subordinated, resigned orientation) was characterized by communicative deficit narratives and conjunctive patterns of resignation, dependency, and restricted agency. Type 2 (reflexive, pro-active orientation) demonstrated communicative acknowledgment of burdens but conjunctive practices of resilience, negotiation, and self-assertion. Unintended pregnancies were frequently constructed as existential crises, in which personal needs and emotions were suppressed. Minority status intensifies these challenges, while ACE -related patterns of behavior were reproduced in the context of pregnancy and intimate relationships.

**Conclusions:**

The study provides valuable insights into the complex realities and decision-making processes of female adolescents with unintended pregnancies. The findings highlight the need for specific support services that strengthen girls’ empowerment and agency in order to promote sexual and reproductive health and rights. Outdated role models that ascribe contraceptive responsibility solely to women should be replaced by gender-equitable sexual education. In addition, legal regulations and counseling services should be better aligned with the often precarious family and partnership situations of young pregnant women.

## Abstract (plain language summary)

**Background:**

The teenage years are an important time for young people to develop their identity and sexuality. Becoming pregnant at this age is often unintended and can be a sensitive and stigmatizing experience. It usually comes with major challenges. This study looks at how teenagers in Germany experience unintended pregnancies, especially when they have faced difficult experiences in childhood (known as Adverse Childhood Experiences, ACE) or violence in relationships (Teen Dating Violence, TDV).

**Method:**

Interviews were analyzed with five young women who experienced an unintended pregnancy as teenagers. These interviews were conducted as part of a larger research project called ELSA, which ran between 2020 and 2024.

**Results:**

The study shows that there are two main ways in which teenage girls deal with unintended pregnancy:

1. The subordinated, resigned type (basic type): These young women often feel powerless, controlled by their environment, and limited in their ability to act.

2. The reflexive, pro-active type (maximum contrast): These young women are able to reflect on their situation, make their own decisions, and act more independently.

The findings also show that traumatic childhood experiences strongly how teenagers cope with pregnancy and how they experience their relationships.

**Conclusion:**

The study highlights the need for specialized support services for teenagers with unintended pregnancies. Such services should strengthen self-determination and agency, replace outdated role models with fairer and more gender-equitable sex education, and ensure that legal regulations and counseling take into account the often difficult family and partnership siuatons of young pregnant women.

## Additional non-English language abstract

**Hintergrund:**

Die Adoleszenz ist eine prägende Lebensphase, in der das eigene Selbstverständnis und die sexuelle Identität entwickelt werden. Ungewollte Schwangerschaften im Jugendalter sind ein sensibles und stigmatisiertes Thema, das häufig mit erheblichen psychosozialen Herausforderungen verbunden ist. Ziel dieser Studie ist es, die Erlebenswelt junger Frauen mit ungewollten Teenagerschwangerschaften in Deutschland zu untersuchen, mit besonderem Fokus auf Adverse Childhood Experiences (ACE) und Teen Dating Violence (TDV).

**Methode:**

Die Analyse basiert auf fünf biografisch-narrativen Interviews die im Rahmen des ELSA-Projekts (November 2020–April 2024) durchgeführt wurden. Das Projekt zielte darauf ab, Einblicke in den Umgang mit ungewollten Schwangerschaften zu gewinnen und Beratungs- und Versorgungsbedarfe abzuleiten. Mittels der dokumentarischen Methode wurde eine sinngenetische Rekonstruktion kommunikativer und konjunktiver Erfahrungen verfolgt, die auf Basis von vier Vergleichsdimensionen kontrastiert wurden: Kindheitsbiografie, Umgang mit der ungewollten Schwangerschaft, Konstruktion der Paarbeziehung und Konstruktion von Minderjährigkeit.

**Ergebnisse:**

Die Analyse zeigt, dass sich über die verschiedenen Vergleichsdimensionen hinweg konsistente handlungsleitende Orientierungen rekonstruieren lassen,. Diese sind durch den Umgang mit Fremdrahmungen geprägt, die entscheidend für die Handlungsfähigkeit und Selbstbestimmung der Jugendlichen sind. Rekonstruiert wurden zwei kontrastive Typen: ein subordinierter, resignierter Typ, der durch Fremdbestimmung und eingeschränkte Handlungsfähigkeit gekennzeichnet ist, sowie ein reflexiver, pro-aktiver Typ, der trotz äußerer Begrenzungen Handlungsoptionen entwickelt und Selbstbestimmung wahrt. Ungewollte Schwangerschaften werden häufig als existentielle Krisen konstruiert, in denen persönliche Bedürfnisse und Emotionen ignoriert oder zurückgestellt werden. Minderjährigkeit verschärft diese Herausforderungen zusätzlich. Die Ergebnisse verdeutlichen, wie traumatische Kindheitserfahrungen (ACE) zu habitualisierten Handlungsmustern werden und wie sich diese Muster im Kontext der Schwangerschaft und in Paarbeziehungen reproduzieren.

**Schlussfolgerung:**

Die Studie liefert wertvolle Einblicke in die komplexen Lebensrealitäten und Entscheidungsprozesse jugendlicher Frauen mit ungewollten Schwangerschaften. Sie unterstreicht die Notwendigkeit spezifischer Unterstützungsangebote, die die Selbstbestimmung und Handlungsfähigkeit der Betroffenen stärken, um die sexuelle und reproduktive Gesundheit und Rechte zu fördern. Veraltete Rollenbilder, die die Verhütungsverantwortung ausschließlich Frauen zuschreiben, sollten durch eine geschlechtergerechte Sexualaufklärung ersetzt werden. Zudem sollten gesetzliche Regelungen sowie Beratungs- und Versorgungsangebote stärker auf die oftmals prekären familiären oder partnerschaftlichen Konstellationen jugendlicher Schwangerer abgestimmt werden.

## Background

Adolescence is a critical phase of life in which personal experiences and decisions are central, and an independent and sexual identity is formed. A key developmental task during this period is to establish a healthy sexual self-concept. This requires a positive and respectful approach to sexuality and relationships as well as pleasurable and safe experience free from coercion, discrimination, and violence [1–3]. Research shows that adolescents form their perspectives on sexual activity, pregnancy and parenthood through reciprocal influences from their environment, including peers, partners, family, community and society in general [4–6]. Family dynamics, social norms, cultural backgrounds, power relations, and poverty have both direct and indirect effects on adolescents’ sexual and reproductive health decisions and behaviors [7–10].

Unintended pregnancy remains a taboo and stigmatized issue, making it difficult for many adolescents to share their experiences. As an unplanned life event, it often develops into an acute and challenging situation that requires rapid decision-making under time constraints. Recognizing one’s needs and boundaries is crucial in order to navigate the situation and assert sexual and reproductive rights. This requires a wide range of resources and support, especially during adolescence, to prevent psychosocial strain, health burdens and long-term socioeconomic disadvantages. So-called “teenage pregnancies” frequently stigmatized, disrupt age-typical development tasks (e.g., separation from the parental home), reinforce dependency and lead to profound life changes [11–14]. Their consequences extend far beyond the pregnancy itself and significantly shape later life trajectories [15–18]. Continuing school or vocational training, for example, becomes a major challenge if the pregnancy is carried to term. Many young mothers drop out of school or training, which often results in financial strain, stigma-related stress and mental health issues, dependence on others, and a precarious long-term life course. In addition to physical and emotional changes, affected teenagers are confronted with complex social and moral challenges such as stigmatization, social exclusion, and violence in relationships, from peers, or from family members [13, 19–21].

Teenage pregnancies, particularly in very young adolescents, are classified as high-risk pregnancies because they often occur before full not accompanied by complete physical, psychological, and social maturity has been reached [11]. This complicates the provision of care, especially as there are no guidelines specifically addressing the needs of affected young women [11]. In 2022, 10,999 babies were born by 15–19-year-old mothers. Although the number of teenage pregnancies has declined in Germany and internationally in recent years [22, 23], unintended pregnancies during adolescence remain particularly challenging due to the interplay of minority status, stigma, and limited resources.

Sexual and reproductive rights include access to health information and services, the right to self-determination in sexual and reproductive decisions, and protection from sexual violence and discrimination [24]. The minority status of affected women is central to understanding unintended teenage pregnancies from a psychosocial perspective. Adolescence is characterized by legal and social ambivalence: on the one hand, adolescents are perceived as immature and subject to parental or institutional authority; on the other hand, they have to make decisions of far-reaching consequence. The debate around reproductive rights reflects these tensions: underaged pregnant women often have limited awareness of their ability to make self-determined decisions about abortion, adoption, or parenthood – especially when in unstable or violent relationships where reproductive coercion may occur [25–27]. These rights apply to all individuals, including minors, and are essential for health and well-being. Institutional, familial and partner-based influences, however, can substantially restrict the available options.

The experience of unintended teenage pregnancies can also be examined through the lens of agency theory and the explanatory model of adverse childhood experiences (ACE). Sexual and reproductive agency refers to the ability of individuals to make and act upon decisions concerning their sexual and reproductive health. This agency is often constrained by social, cultural and legal norms, and adolescents are particularly affected due to the legal framework [28–30]. ACE – such as abuse, neglect, and/or family dysfunction – not only increase the likelihood of unintended pregnancies, but also shape psychosocial development, agency, decision-making, and adolescents’ sense of self-efficacy. They further influence coping strategies during and after an unintended pregnancy [31–34].

A particularly vulnerable group for both teenage pregnancies and the experience of ACE, past or present, are adolescents involved in violent relationships (= teen dating violence; TDV). In Germany, about two of three teenagers report at least one experience of partner abuse [35]. These adolescents face a significantly higher risk of becoming pregnant as minors compared to peers without such experiences [36, 37], and many continue to face violence even during pregnancy [35, 38]. Previous studies also suggest a close relationship between ACE and TDV victimization [39, 40], with sexual abuse, interparental violence and parent mental illness showing the strongest associations [41].

To date, however, only a few recent studies have focused specifically on the vulnerable group of unintentionally pregnant minors, particularly in Germany [11, 27, 42–44]. To our knowledge, no previous study has examined teenage pregnancies by considering ACE and TDV in combination as experiential contexts that shape adolescents’ meaning-making and coping processes. This article addresses this research gap and seeks to provide exploratory insight into unintended pregnancies in adolescents as a biographical event. It aims to answer two guiding questions: (1) What are the central aspects of experiencing an unintended teenage pregnancy? (2) How is unintended pregnancy in adolescence constructed and how are modes of its processing?

Based on five biographical-narrative interviews, a sense-genetic reconstruction of conjunctive experiences is pursued using the documentary method [45]. The aim is to gain a deep understanding of the experiences of those affected, not only to contribute to the scientific discussion, but also to develop practical implications for the support and counseling of this vulnerable group, and thus to approaches for strengthening their sexual and reproductive health and rights.

## Methods

The data stem from the project *ELSA* (01.11.2020–30.04.2024), which aimed to gain insights into the experience and management of unintended pregnancies in order to identify counselling and care needs [46]. In addition to the quantitative survey, narrative-biographical interviews [47] were conducted to obtain in-depth information on the diverse life situations of women with unintended pregnancies. These interviews were part of the sub-project on so-called vulnerable groups (ELSA-VG), carried out between January and November 2022 at the University of Applied Sciences Nordhausen. This sub-project specifically focused on women who had experienced intimate partner violence (IPV) or have a history of migration. An open narrative stimulus was used, beginning with childhood and continuing to the current life situation and then immanent and exmanent questions were asked about decision-making process regarding the further carrying of the pregnancy and the medical and psychosocial care situation [46].

Participants were recruited through the quantitative representative survey of the joint project, in which respondents could indicate at the end of the questionnaire. whether they were willing to take part in a narrative interview. Recruitment was also conducted in multiple languages via public institutions, counseling centers, websites, and social media. To ensure easy accessibility and due to the pandemic-related restrictions, the interviews were mainly conducted digitally.

In total, 52 interviews were conducted: 25 interviewees had experienced IPV, 21 had a history of migration, and among 6 cases both aspects occurred. For the analysis of unintended pregnancies in adolescence, we focused on five of the narrative-biographical interviews, were selected based on specific sample characteristics: all five women had experienced an unintended pregnancy as teenagers, adverse childhood experiences (ACE), and intimate partner violence.

### Data analysis

To gain an initial impression of key topics, the interviews were coded using content analysis, with two researchers independently applying a double-coding procedure. Subsequently, the documentary method [48] was employed to analyze the interviews in greater depth and to reconstruct the guiding orientations of female adolescents with unintended pregnancies.

The documentary method is rooted in the praxeological sociology of knowledge (Bohnsack). Its central epistemological principle lies in the differentiation between two forms of knowledge: communicative knowledge and conjunctive knowledge [45].

Communicative knowledge refers to explicit, reflexively accessible, and generalized knowledge expressed through concepts, norms, and role expectations. It represents the level at which actors relate to one another in socially institutionalized ways. Typical examples include explicit argumentations, normative attributions, or justifications that are intelligible across different contexts and can be intersubjectively validated, independent of individual experience.

In contrast, conjunctive knowledge is understood as implicit, atheoretical experiential knowledge. It is embedded in collective experiential spaces and is accessible only to those who share these contexts. This knowledge is not acquired through conceptual reflection but through joint practice, habitual actions, and collectively shared experiences. It manifests itself in the performance and modus operandi of social practice and guides action without requiring explicit articulation. Mutual understanding is achieved on the basis of shared experiences within the medium of what is taken for granted, which is why conjunctive knowledge has also been described as tacit knowledge [49]. This experiential knowledge constitutes the orientation framework in the narrower sense.

The documentary method analytically addresses precisely this tension between communicative and conjunctive knowledge. It is in this space – where individuals must relate societal demands, normative expectations, and habitual orientations – that coping challenges arise. These coping processes can be interpreted as documents of how actors construe and manage their biographical and social tensions (orientation framework in the broader sense). Methodologically, the reconstruction therefore focuses not only on the manifest content of statements (the “what”), but above all on the orientation frameworks that become visible in the performance of narration, action, and interaction (the “how”) [50, 51].

The concept of the ‘orientation problem’ refers to the central biographical and social challenges in which actors must negotiate between external framings and their habitual orientations. It is not a predefined category but emerges from the reconstructive analysis of how participants produce meaning and cope with crises in their narratives. In this study, the orientation problem was approached across four comparative dimensions: (1) the processing of childhood biography within the family of origin, (2) the handling and decision-making process regarding the unintended pregnancy, (3) the construction of the intimate relationship, and (4) the construction of minority.

Because unintended pregnancy is a taboo and stigmatized topic, many adolescents feel burdened when talking about their experiences. The documentary method is therefore particularly suitable for interpreting data beyond what is explicitly said and for accessing hidden structures of meaning and knowledge. The researchers took an active role in the process of analyzing and producing knowledge engaging in joint discussion, debate, and reflection on assumptions and interpretations. A sensitive approach was ensured throughout the intensive examination of the data.

In practice, the action orientations of a case were first reconstructed and then linked to other cases on the basis of their structural similarities. Through minimum and maximum contrasting comparisons, a generalizable typology was developed. Type information was initially carried out in a sense-genetic manner and later sociogenetically. For ensure anonymization, case-specific information, such as precise age details was generalized. These changes are indicated by square brackets, e.g. “I was 15 years old” in “I was [under 16 years old]”. The developed types provide insights into the socio-cultural conditions and contexts in which specific orientations are produced in the experience of unintended teenage pregnancies.

## Results

The reconstructive analysis of the five biographical-narrative interviews reveals two contrasting orientation frameworks (in the broader sense) across four comparative dimensions: (1) the processing of one’s childhood biography within the socialization context of the family of origin, (2) the dealing and decision-making process regarding the unintended pregnancy, (3) the construction of the intimate relationship, and (4) the construction of minority. In line with the logic of the documentary method, a distinction is made between communicative knowledge – social identities, process of social identification, and common-sense theories – and conjunctive knowledge – habitus as the modus operandi of social practice (Bohnsack 2021). the tension between communicative and conjunctive knowledge. It is precisely in this interplay that it becomes visible how adolescents construct and cope with unintended pregnancy against the background of minority status.

From this interaction, two contrasting types were reconstructed:

- Type 1 (basic type): subordinated, resigned type (restricted in agency).
- Type 2 (maximum contrast): reflexive, pro-active type (capable of agency).

These types are not to be understood as fixed attributes of individuals. Rather, they are ideal-typical abstractions that illustrate how adolescents, within a constitutive external framing (minority and unintended pregnancy), discursively articulate and practically process the tension between normative expectations and habitualized practices.

An overview of the case allocation for the two types, including the specific sample characteristics (outcome of unintentionally pregnant as teenagers, ACE and TDV), is shown in Table 1.

**Table 1:** Overview of the case allocation of the typologies including the specific sample characteristics (ACE, TDV and outcome of unintentionally pregnant as teenagers)

Types	Cases	Age interview period	Highest degree at time of focus pregnancy	ACE	TDV	Unintended pregnancy as teenager (pregnancy outcome)
Type 1 (basic type) “the subordinated, resigned type “ Mode of processing: - Passivity - Dependency - Resignation - Perseverance	Sarah Noack (P18)	35	None	**Abuse**: emotional **Household dysfunction**: violent mother, alcohol abusing father, parental separation/divorce **Neglect**: emotional	Psychological and emotional violence; physical violence (e.g., slapping) Coercive control (to abort – although no longer possible)	pregnancy was carried to term (detection in the 4th month)
	Nicole Graf (P17)	28	None	**Abuse**: emotional **Household dysfunction**: mother with mental illness, parental separation/divorce **Neglect**: emotional	Psychological and emotional violence (e.g., insults, threats to leave, suicide threats, blame); physical violence (e.g., slapping, shoving)	pregnancy was aborted
	Alexa Schröder (P15)	28	None	**Abuse**: emotional **Household dysfunction**: mother with mental illness **Neglect**: emotional	Psychological and emotional violence; Coercive control (to abort)	two unintended pregnancies as teenager with same partner; both pregnancies were aborted
	Nadine Walter (P11)	34	None	**Abuse**: emotional, physical, sexual **Household dysfunction**: parental separation/divorce **Neglect**: emotional, physical	No information available (regarding relationship during unintended teenage pregnancy)	pregnancy was carried to term (detection in the 4th month)
Type 2 (maximum contrasting) “reflexive, pro-active type” Mode of processing: - Rationality - Self-determination - Reflection	Lina Schubert (P7)	24	Secondary school degree (intermediate level)	**Household Dysfunction**: parental separation/divorce	Psychological and emotional violence (e.g., insults); physical violence (shoving)	pregnancy was carried to term

### External framing as a tertium comparisons and common basis of experience

All five cases conceptualize the unintended pregnancy in the context of minority as an external framing [52]. This framing constitutes the tertium comparationis that connects all cases on an abstract level: it reflects the structural power of institutions (e.g., health services, child protection), legal regulations, social norms, and socio-economic dependencies within the family. This invisible power limits adolescents’ scope of action and frames their decision-making processes, thereby demonstrating the productive character of power. What differentiates the two reconstructed types is the way this external framing is performatively processed on communicative and conjunctive levels. Contrastive sense-genetic typology across comparative dimensions.

In the following, the reconstructed types are contrasted along the four comparative dimensions to illustrate that both types share the same orientation problems, yet differ fundamentally in how they process them (“contrasts within commonalities”). Table 2 provides an overview of the sense-genetic typology across comparative dimensions.

**Table 2:** Overview of the sense-genetic typology with comparative dimensions

Orientation problem	Type 1: subordinated, resigned (restricted in agency)	Type 2: reflexive, pro-active (capable of agency)
Childhood biography	Orientation towards deficits, vulnerability	Orientation towards resources
Unintended pregnancy	Experience of degradation, repression of emotions	Decision-making oriented towards own needs and relevance structures
Intimate relationship	Discrepancy between positively constructed discourse and performative experience of dependency/subordination	Integrative, but distancing when facing violence
Minority	Experience of restriction, fear, and powerlessness	Simultaneous experience of vulnerability and agency
Cases	P11, P15, P17, P18	P7

### Comparative dimension 1 - childhood biography in the socialization context of the family of origin

The family emerges as a central experiential space in which basic orientations of trust, support, and agency – or conversely of blame, devaluation, and powerlessness – are produced. The childhood biographies of all interviewees are marked by experiences of dysfunction and conflict, partly including abuse and neglect. While this external framing constitutes a common basis for all cases, differences become apparent in how these experiences are communicatively framed and conjunctively processed.

#### Type 1: Communicative knowledge

Childhood is framed as unstable and deficit-oriented, shaped by blame and neglect. Nadine recalls:

*“And their father [of the stepsiblings] always touched me like that. And then my mother always said: ‘Yes*,* it’s your own fault*,* you don’t have to walk around here in short clothes.’ So*,* she always accepted it like that”* (Nadine, P11, Pos. 3). Alexa adds: *“And she always said*,* yes*,* you’ll never amount to anything. […] I was bullied because I was very*,* yes*,* reserved and not self-confident”* (Alexa, P15, Pos. 5).

#### Conjunctive knowledge

Patterns of resignation and fatalism emerge. Sarah reports: *“And my mom then never got in touch again*,* she just didn’t want us to go to dad. […] The fact that my dad was simply taken away from me was very difficult for me … […]. Because I was still very young”* (Sarah, P18, Pos. 3–5). Nicole recalls: *“My dad often shouted at my mom. My mom didn’t fight back and just cried. Yes. And that somehow got worse and worse. Especially when I started doing so badly at school”* (Nicole, P17, Pos. 3–10). These accounts point to a habitualized mode of “enduring” and subordination.

#### Type 2: Communicative knowledge

Lina also describes familial dysfunction but frames it reflexively:

*“Exactly*,* when I was a teenager*,* my mother and stepfather separated physically. And that was very*,* very difficult for me”* (Lina, P7, Pos. 3). At the same time, she emphasizes supportive structures: *“Well*,* I have to say that my grandma is a bit closer to me than my mother*,* because*,* I don’t know*,* she’s always there for me”* (Lina, P7, Pos. 3).

#### Conjunctive knowledge

She translates crises into active coping: *“I then said of my own accord*,* okay*,* I’ll look for another apartment*,* but it really got to me”* (Lina, P7, Pos. 5). Here, habitualized trust in one’s own capacity to act becomes visible.

#### Orientation framework

Type 1 communicative knowledge frames childhood as victimization and conjunctive knowledge processes it through resignation and subordination.

Type 2 communicative knowledge acknowledges burdens, while conjunctive knowledge processes them through pro-active engagement and resource mobilization.

### Comparison dimension 2 - dealing with and decision-making about unintended pregnancy

Unintended pregnancy is experienced in all cases as a turning point and biographical rupture, associated with uncertainty, stigmatization, and limited options for action. Thus, it constitutes a central orientation problem that must be negotiated at the intersection of individual needs, normative attributions, and institutional expectations. While external framing by societal norms forms a common starting point, cases diverge in their communicative and conjunctive processing.

#### Type 1: Communicative knowledge

Pregnancy is framed as a crisis, norm violation, and external framing. Sarah recalls:

*“And he was also strictly against it*,* he also told me to have an abortion. And otherwise*,* the relationship is over”* (Sarah, P18, Pos. 5). Nicole remembers the stigmatizing reaction of a medical assistant: *“So the doctor’s assistant called me and my boyfriend into the lab and asked why I thought I wasn’t getting my period. Then I said: ‘Yes*,* I don’t know*,* maybe I’m pregnant’. And then she said: ‘Yes*,* congratulations*,* you’ve done really she said’”* (Nicole, P17, Pos. 3).

#### Conjunctive knowledge

These framings translate into feelings of helplessness and repression of personal needs. Nicole: *“It was like a switch flipped in my head. […] Okay*,* what do I have to do now*,* bang*,* bang*,* bang*,* to get out of it”* (Nicole, P17, Pos. 146). Nadine adds, referring to her late diagnosis: *“Yes*,* there’s nothing more you can do. You have to have the child”* (Nadine, P11, Pos. 43). Nicole further highlights her sense of existential crisis: *“And for me it was somehow clear that if I had this child*,* then I would not only be pregnant [under age of 16] without a school-leaving certificate and without a boyfriend*,* something*,* but I would also be homeless. […] I just saw myself facing absolutely nothing.”* (Nicole, P17 pos. 164).

#### Type 2: Communicative knowledge

Lina frames the decision to continue her pregnancy as self-determined, enabled by familial support: *“For me*,* as soon as I knew I was pregnant*,* I knew straight away I was going to have it. Because I simply have the support of my family”* (Lina, P7, Pos. 3).

#### Conjunctive knowledge

She reflects on her options and positions herself as capable of making her own decision: *“Well*,* I made that decision directly for myself because I simply couldn’t do it*,* I couldn’t say*,* no*,* I’ll have an abortion. […] Not everyone has to be able to do that”* (Lina, P7, Pos. 3). Here, abortion is constructed as a negative counter-horizon – not a real option, but an empirical comparative frame.

#### Orientation framework

Type 1 communicative knowledge constructs unintended pregnancy as external framing and norm violation, while conjunctive knowledge processes it through helplessness, repression of personal needs, and existential crisis.

Type 2 communicative knowledge reflects possible alternatives and frames the decision as self-determined, while conjunctive knowledge processes it reflexively, enabling the adolescent to assert her own needs against external expectations.

### Comparison dimension 3 - construction of the couple relationship

The intimate relationship constitutes a central point of reference in all cases, where security, belonging, and emotional stability are sought. Common to the interviewees is that partnerships are brought up as an important resource in the transition out of the family of origin – often associated with the hope for protection and support. At the same time, however, relationships also represent orientation problems: while they are discursively constructed as a longed-for place of refuge, they are simultaneously experienced as a source of conflict, violence, and dependency. Against this shared background, clear differences emerge in how they are communicatively framed and conjunctively processed.

#### Type 1: Communicative knowledge

Relationships are constructed as positive, emotionally supportive, and oriented toward stereotypical gender roles. Sarah notes: *“So he always went to work. […] Back then*,* it was like that*,* he goes to work*,* everything is fine”* (Sarah, P18, Pos. 5). Alexa recalls: *“I never wanted to be at home […] I felt comfortable with him”* (Alexa, P15, Pos. 18).

#### Conjunctive knowledge

However, they are lived as spaces of subordination and dependence, shaped by violence and ultimatums: *“Either you have an abortion now or I leave you”* (Sarah, P18, Pos. 37–39). Alexa describes how she subordinated her own needs: *“Then I said at some point*,* okay*,* I’ll have another abortion*,* then we can be happy”* (Alexa, P15, Pos. 7–8).

#### Type 2: Communicative knowledge

Lina integrates her partner into her family context: *“I always felt that my family was also his family”* (Lina, P7, Pos. 3).

#### Conjunctive knowledge

However, she responds to violence with active distancing: *“When he once went off on me […] I pushed him away […] and thought*,* I definitely have to pull the ripcord now”* (Lina, P7, Pos. 3). Thus, she habitually enacts boundaries rather than subordination.

#### Orientation framework

Type 1 communicative knowledge frames relationships in positive, stereotypical terms, while conjunctive knowledge processes them as dependency and subordination.

Type 2 communicative knowledge integrates the partner into family structures, while conjunctive knowledge processes experiences of violence through distancing and resistance.

### Comparative dimension 4 - construction of minority

Minority status constitutes a central external framing for all interviewees, fundamentally shaping their decision-making scope. All cases construct minority as a condition of dependence, vulnerability, and limited autonomy, yet their communicative framings and conjunctive practices differ.

#### Type 1: Communicative knowledge

Minority is constructed as dependence and limitation of action: *“Whether I should just have an abortion without them knowing*,* which is of course difficult [as a teenager]”* (Alexa, P15, Pos. 7). Nadine reports her mother’s derogatory comment: *“But then my mother*,* when the [episiotomy after birth] was to be stitched again*,* she didn’t agree to me getting the anesthetic. It had to be done without anesthesia. Then she kept saying: ‘Yes*,* anyone who can have a child at such a young age can bear it now. And I hope you never have children again.’ So that was really bad for me.”* (Nadine, P11, Pos. 3). Nicole highlights the stigmatization she experienced in a medical setting: *“And well*,* this doctor greeted me by saying: ‘You’re the youngest person I’ve had this year’. That’s great. Yes*,* exactly. And he was just teasing me the whole time. About whether I’m too stupid to use contraception and things like that.”* (Nicole, P17 Pos.3). Sarah further points to the paradox of minority and motherhood: *“You may be a mother*,* but you are also a child.”* (Sarah, P18 pos. 15). She also describes the incompatibility of young motherhood with education: *“I had to drop out of school in the seventh grade […] But when I had my daughter*,* I tried again and again to enroll in school*,* to catch up on something so that I could get ahead. Yes*,* but I had to stop again and again because I no longer had a babysitter.”* (Sarah, P18 pos. 5).

#### Conjunctive knowledge

Fear and powerlessness dominate: Sarah describes her constant fear of losing her child (*“I was always afraid that they would take my child away from me”*, Sarah, P18, Pos. 26–27). She further emphasizes the sense of being left without support when institutions repeatedly referred her back to her dysfunctional home: *“You don’t have an apartment. No matter what*,* they kept saying: ‘Yes*,* you have to go home. You have to go home. Your mother gets money for you.’ Yes*,* but what if it doesn’t work at home? So*,* you were already lost again. Because nobody helped you anyway”* (Sarah, P18, pos. 26–27). Nadine underline not being heard: *“It was really bad that no one listened to the [between 10 and 20]-year-old*,* only to the adult”* (Nadine, P11, Pos. 7).

#### Type 2: Communicative knowledge

Lina constructs minority ambivalently: as vulnerability but also as an opportunity for support: *“We had virtually no prospects*,* but my family still supported me”* (Lina, P7, Pos. 3). She also emphasizes the positive developmental aspects of young motherhood: “*Well*,* you always say that you grow with your experiences when you have a child somehow […] And yes*,* well*,* yes*,* I have to say that has really*,* really encouraged me and I am also very proud to be a mother.”* (Lina, P7 pos. 9).

#### Conjunctive knowledge

She positions herself self-confidently: *“I said*,* hey guys*,* I’m young*,* of course I have a child*,* but that doesn’t mean I’m not allowed to do anything here anymore […] So*,* yes*,* I’ve always had the attitude that I’m going to do my thing and not let anyone talk me into it.”* (Lina, P7, Pos. 13). Here, minority is reframed into a horizon for agency.

#### Orientation framework

Type 1 communicative knowledge constructs minority as limitation, subordination and stigmatization, while conjunctive knowledge processes it through fear, helplessness, and institutional disempowerment.

Type 2 communicative knowledge acknowledges vulnerability but reframes young motherhood as a positive developmental horizon, while conjunctive knowledge processes it through resilience and proactive self-assertion.

### Typology – subordinated, resigned vs. reflexive, pro-active orientation

Across all cases, the external framing of unintended pregnancy in adolescence (tertium comparationis) emerges as the shared experiential basis. Yet the ways in which communicative and conjunctive knowledge interact differ fundamentally.

- *Type 1 (subordinated, resigned orientation)*: Communicative deficit narratives, external attributions, and stigmatization are translated into conjunctive patterns of resignation, fear, and subordination. Adolescents construct themselves as dependent on external decision-making, suppress their own needs, and reproduce heteronomy.
- *Type 2 (reflexive, pro-active orientation)*: Communicative burdens are acknowledged but reframed conjunctively into resilience, negotiation, and self-assertion. Adolescents construct themselves as actors of their own biographies, capable of developing and pursuing courses of action despite normative constraints.

While minority status intensifies orientation problems in both types, the processing differs: Type 1 reproduces subordination and helplessness, whereas Type 2 reflexively transforms external framings into opportunities for self-determination.

## Discussion

The empirical analysis shows that homologous action-guiding orientations can be reconstructed across the comparative dimensions. These orientations manifest both in communicative knowledge (explicit, normative and reflexively accessible schemata) and in conjunctive knowledge (implicit, routinized modes of practice and habitualized patterns). They have a lasting impact on adolescents’ life paths and decision-making processes, as they are incorporated into action routines. The tension between these two forms of knowledge illustrates how adolescents discursively and interactively negotiate the discrepancy between common-sense theories, normative expectations (e.g., regarding family, partnership, or motherhood), and embodied action routines acquired during childhood.

The orientations reconstructed in this study are consistently shaped by how young women deal with external framings—institutional, legal, social, and relational influences that delimit or expand their scope of action. Two contrasting orientation types were reconstructed: the *subordinated*,* resigned* type and the *reflexive*,* pro-active type*.

These differences highlight the need to develop targeted support services that strengthen adolescents’ self-determination and agency while also addressing their often precarious living conditions. What becomes visible is not only how adolescents cope with unintended pregnancies, but also how they discursively frame them – for instance, as norm deviation, existential crisis, or biographical disruption – and how these framings are subsequently translated into conjunctive, habitual practices. Particularly relevant is how the external framing of minority status and unintended pregnancy is processed differently across the two types: while Type 1 largely reproduces external power structures and constructs itself as subordinated (restricted in agency), Type 2, despite normative constraints, reflexively develops and asserts alternative courses of action. These findings underline how decisive external framings are for adolescents’ capacity to act (self-efficacy) and for their sexual and reproductive agency. The tension between normative expectations (communicative level) and embodied practices (conjunctive level) makes visible the orientation problems adolescents face. Coping is not merely an individual psychological matter but also a social and biographical negotiation of contradictory demands. In this sense, external framings and orientations must be seen as mutually constitutive: framings shape available options and practices, while orientations guide how these framings are interpreted, processed, and enacted. This relationship is best understood as dynamic and reciprocal rather than as a one-directional causal mechanism.

It is therefore crucial to strengthen support systems and social networks to expand adolescents’ autonomy and reinforce their agency. In this context, resilience factors must be considered in relation to adverse childhood experiences (ACE), particularly the development of autonomy, competence, and relatedness in early socialization and their role in decision-making regarding unintended pregnancies [53–55].

Being underage intensifies the challenges and restrictions that adolescents face in dealing with unintended pregnancies. Adolescence is characterized by legal and social ambivalence: while teenagers are often expected to make far-reaching reproductive decisions, they are simultaneously positioned as immature and dependent. Social norms and structural conditions contribute to adolescents perceiving themselves as limited in their agency and externally patronized. Developing sexual and reproductive health literacy through early and targeted information and counseling can empower adolescents, encourage them to claim their rights, and strengthen sexual and reproductive agency. Diverging views and opaque legal frameworks, particularly in Germany, exacerbate the perception of unintended pregnancy as crisis-ridden and stigmatized. Our findings also show that such external framings are not only discursively reproduced but are incorporated into habitualized practices, reinforcing either subordination (Type 1) or enabling reflexive negotiation (Type 2).

The interviews revealed that adolescents predominantly reported their experiences in an experiential mode. Analytical access was therefore primarily situated at the level of conjunctive knowledge – in habitualized practices, routines, and performative ways of coping with external framings – rather than at the level of communicative knowledge, which would have involved more theoretical reflection or conceptual articulation. A theoretically elaborated perspective on “unintended pregnancy in adolescence” was largely absent; instead, performative experiential dimensions prevailed. This is not only methodologically relevant but also crucial for practical implications: support services must address this level of habitualized, embodied practice and cannot rely solely on normative discourses or explicit forms of knowledge.

Drawing on Becker’s interactionist deviance theory “Outsiders” teenage pregnancy can be interpreted as a social norm deviation. What is perceived as deviant behavior is characterized by two elements: the breaking of social rules and the definition of this act as deviant by the relevant social group. Thus, deviance is not inherent in the act itself but emerges from social processes of rule-setting and sanctioning. This perspective is particularly relevant for the subordinated, resigned type, where stigmatizing framings are not only imposed externally but also internalized and reproduced in habitualized orientations [56, 57].

While this perspective primarily concerns the young women in our sample, teenage pregnancies also affect boys in their role as young fathers, even though this perspective was not part of our study design. Our focus was explicitly on the lived experiences of adolescent girls, as they are directly confronted with the embodied reality of pregnancy and birth. Nevertheless, gendered expectations and stereotypes shape not only girls’ but also boys’ orientations and vulnerabilities, highlighting the importance of future research that systematically includes both perspectives.

Social external framing through norms, values and legal provisions therefore constitute an elementary component of stigmatization and discrimination [21]. In this context, the decriminalization of abortions under § 218 StGB in Germany can be seen as a long-needed step to reduce normative fears, counteract stigmatization, and create conditions for self-determined reproductive rights [58, 59]. However, our results highlight that such legal reforms must be accompanied by broader support structures that address both the communicative framings (e.g., social stigmatization, moral attributions) and the conjunctive orientations (e.g., resignation, fear, or, alternatively, reflexive self-assertion) of adolescents.

The women in this sample share experiences of vulnerability rooted in unstable or precarious family relationships (“broken homes”). The absence of supportive family environments fosters heteronomy and restricted agency. Even as adolescents and later as adults, these orientations continue to be reproduced, for example in the tolerance of violent relationships. This corresponds to the subordinated, resigned type, where communicative framings of deficit and guilt are coupled with conjunctive practices of endurance, fear, and subordination. In contrast, the reflexive, pro-active type demonstrates that supportive family relationships and resources can counterbalance these trajectories, enabling resilience and self-assertion. These findings are in line with the overarching results of the ELSA-VG study [46]. and with existing research showing that violence experienced in childhood is one of the strongest predictors of later IPV [60, 61].

During childhood, affected persons often learn that love and violence are intertwined, a dynamic that is then reproduced in later relationships. Violent partners frequently externalize responsibility by attributing blame to the women, which is internalized and incorporated as habitual practice. This mechanism is particularly evident in Type 1, where communicative framings of guilt and stigma are homologously translated into conjunctive orientations of self-blame and subordination. Type 2, however, shows that this homologous reproduction can be interrupted when adolescents are embedded in stabilizing and supportive relational contexts (see also [46, 62]).

Alongside these conjunctive patterns of endurance and self-blame, the data also reveal counter-horizons: communicative knowledge about “how things should be” – for example, expectations of a caring partnership or supportive family – is invoked but often clashes with lived reality. It is precisely in this dissonance that the documentary method enables us to trace how adolescents attempt to reconcile normative ideals with habitual practices.

Social support thus emerges as a crucial protective factor [63, 64]. Adolescents who have experienced ACE such as abuse, neglect, or dysfunctional family relationships are more likely to have unintended pregnancies. These experiences not only shape psychosocial development but also restrict reproductive agency. In Type 1, ACE foster orientations of dependency and helplessness; in Type 2, however, such experiences can be reframed through supportive networks into reflexive coping and active negotiation [65–67]. ACE therefore undermine adolescents’ confidence in their agency and decision-making capacity but simultaneously provide the backdrop against which different conjunctive coping strategies emerge: resignation and subordination on the one hand, or resilience and negotiation on the other.

These findings underline the importance of trauma-informed care. Professional actors must be able to recognize when adolescents’ communicative accounts of burden (e.g., guilt, stigma) are linked to conjunctive practices of fear, avoidance, or resignation. Support services should therefore aim not only at correcting “knowledge gaps” on the communicative level, but also at intervening in the conjunctive dimension by fostering resilience, trust, and embodied practices of self-assertion.

### Strengths and limitations

This study offers in-depth insights into the experiences of adolescents who became unintentionally pregnant. The use of biographical-narrative interviews provides a unique methodologically access to case reconstruction and to adolescents’ orientations, enabling conjunctive experiences to be traced across childhood and adolescence in different social contexts.

A particular strength lies in the application of the documentary method, which goes beyond manifest content analysis and allows for the distinction between communicative and conjunctive knowledge. By reconstructing both explicit normative references (communicative schemata) and implicit habitual orientations (conjunctive experience), the study reveals how adolescents navigate the tension between societal expectations and their own embodied practices.

The analyzed subsample is relatively small due to the specific focus on cases characterized by multiple vulnerabilities (ACE and TDV). This limits the generalizability of the findings enables a particularly dense reconstruction of orientation frameworks. Through sense-genetic analysis, homologous performative productions and shared conjunctive experiences could be reconstructed across cases, visible in both positive and negative (counter-)horizons. The reconstructed basic type was contrasted with one maximum-contrast case. While this strengthens the typological analysis, it cannot be ruled out that additional cases of the second type or further subtypes could be identified in larger samples. Nevertheless, this typological construction provides valuable first insights into the socio-genetic tendencies underlying the orientations of young women in the context of unintended pregnancies.

As all analyzed cases were marked by ACE and experiences of TDV, the results cannot be generalized to all teenage pregnancies. However, this focus on a particularly vulnerable group makes the findings especially relevant for understanding the interplay between biographical vulnerability, external framing, and reproductive agency. It also shows how ACE contribute to the development of habitual practices that are reproduced in the way unintended pregnancies are processed. The results provide important entry points for understanding unintended pregnancies in adolescence under vulnerable living conditions, which can be elaborated further in future analyses.

The use of the documentary method not only enabled an in-depth reconstruction of the social realities and patterns of action of those affected but also revealed how adolescents confront external framings within their social practice. The explicit consideration of ACE highlights the profound effects of traumatic childhood experiences on teenagers’ agency and their decision-making processes. This perspective broadens the understanding of the long-term consequences of childhood trauma and its impact on reproductive health and agency.

Although this study refers to a specific geographical and social context in Germany, it is likely that the reconstructed sense-genetic types can also be identified in international contexts, provided that women construe unintended pregnancy as an external framing and have experienced ACE. Future research should therefore explore the transferability of these findings through comparative, transnational studies and investigate how different institutional or cultural contexts shape the interplay between ACE, external framing, and reproductive agency.

### Implications for practice

The results of this study offer valuable insights into the living conditions and decision-making processes of teenage girls with unintended pregnancies in the context of multiple vulnerabilities. A first implication concerns the need to systematically assess experiences of violence in counseling and care. Previous studies, including the WHO multi-country Study on Women’s Health and Domestic Violence against Women, have shown that unintended pregnancy and abortion can serve as important indicators of intimate partner violence [68]. Our findings confirm that teenage pregnancies are often embedded in violent or coercive relationships, making it essential that professionals screen for possible victimization as an initial step when providing support.

A second implication concerns sexual education. While early and appropriate education about contraceptives and the consequences of unprotected intercourse is widely recognized as central to prevention [69–73], current curricula often remain limited. They frequently emphasize consensual, safe, and pleasurable experiences, but tend to neglect the biological and physiological dimensions of fertility, the differences in sexual drive and hormonal development, as well as gender-specific vulnerabilities and unequal dynamics of sexual relationships. This lack of comprehensive knowledge restricts adolescents’ freedom of choice and their ability to make informed decisions.

Our findings support research highlighting the importance of sexual literacy – including knowledge of anatomical and physiological changes, reproductive cycles, and fertility awareness – as a protective factor against teenage pregnancy. Evidence from Colombia demonstrates that adolescents who had received information on body functions and pubertal changes showed significantly lower pregnancy prevalence. Therefore, sexual education should not only focus on consent and relational aspects but also integrate fertility awareness and body knowledge as crucial dimensions of reproductive health literacy. This dual approach could help adolescents – especially young girls –develop both the discursive resources (communicative knowledge) and the embodied awareness (conjunctive knowledge) necessary for agency in reproductive matters [74]. In addition, sexuality education must also include the ability to perceive boundaries and to say “no”. Equally important, boys should be taught to respect the boundaries of their partners and to act responsibly in intimate relationships. At the same time, sexual education should address the age of consent more explicitly. This is not only crucial for preventing sexual exploitation but also for enabling adolescents to understand the legal and social boundaries of consensual relationships. Integrating such content aligns with the guidelines of child protection agencies (e.g., ECPAT International) and supports adolescents in navigating their rights [75].

Furthermore, interventions should move beyond attributing contraceptive responsibility primarily to girls and embed shared responsibility with boys from an early age. This shift is necessary to overcome outdated gendered norms that continue to promote “victim blaming” in the areas of contraception, consent, and violence [76]. Strengthening reproductive rights, promoting gender equality, and overcoming transgenerational inequalities therefore requires integrating male adolescents into preventive approaches and dismantling stereotypes and gendered role attributions from an early age.

Beyond, the results underline the need to develop and expand specific support and counseling services that take into account the individual needs and life situations of those affected, in order to promote the agency and self-determination of underage women in the context of unintended pregnancy and potentially young motherhood. Traumatic childhood experiences, e.g. dysfunctional, unstable family relationships as well as toxic and violent partnerships should be systematically considered in counseling settings, and sensitivity to such vulnerabilities should also be strengthened in outreach and open youth work. Due to shame, guilt and fear, ACE may be downplayed or concealed [26]. Professional actors therefore require trauma-informed training to identify signs, create safe spaces, and provide adequate support. Trauma-informed care represents a particularly appropriate framework, as it fosters awareness, knowledge, and skills regarding trauma, while promoting safe, trustful, and empowering environments. At the same time, interventions must not only address the systemic level but also extend across generations. For example, more attention needs to be focused on children of mothers affected by violence in order to end the cycle of transgenerational violence [77–79].

Finally, the legal framework for unintentionally pregnant underaged women currently requires the involvement or consent of parents or legal guardians at several points. Our findings show that this can be problematic, especially when the family context is precarious, unstable, or even harmful. Counseling and care services should therefore prioritize the wishes and needs of minors themselves and, where necessary, enable individualized arrangements that safeguard their reproductive rights and protect them from further harm. The possibility for adolescents to circumvent parental consent through court procedures is usually insufficient to prevent negative consequences such as delayed and/or unsafe abortions or an unwanted continuation of a pregnancy. A reform of legal and procedural frameworks is needed to ensure timely, safe, and truly self-determined reproductive decisions for adolescents.

## Conclusions

Overall, the analysis shows that unintended pregnancies in adolescence are associated with complex and multi-layered challenges that are negotiated at the intersection of normative expectations (communicative knowledge) and habitual practices (conjunctive knowledge). These challenges are further intensified by the constitutive external framing of minority status, which structurally limits the scope of action available to adolescents. The reconstructed types – one characterized by subordinated, resigned and other by a reflexive orientation – illustrate how young women position themselves in relation to external framing such as family, partnership, institutional setting, and legal structures. While all cases share the experience of external framing as a common basis, their processing diverges: for some, this results in resignation, dependency, and restricted action horizons, whereas in one, external framings are actively negotiated and self-determination is maintained.

These findings underline that unintended pregnancy in adolescence cannot be reduced to individual decision-making alone but must be understood as a process shaped by structural constraints, normative constructions, and embodied orientations. Consequently, there is a strong need for individualized and context-sensitive support services that address both the discursive constructions and the habitual practices of young women. Strengthening and diverse support systems can help to expand adolescents’ scope for action, reinforce their agency, and promote the sexual and reproductive health and rights of underaged women.

## Data Availability

Due to the sensitive nature of the data and ethical restrictions related to the protection of participants victimized by IPV, the datasets generated and analysed during the study are not publicly available. Aggregated or anonymized data may be provided by the corresponding author upon reasonable request and in accordance with ethical approval.

## Abbreviations

ACE: Adverse Childhood Experiences
TDV: Teen Dating Violence
IPV: Intimate Partner Violence
ELSA: Experiences and Living Conditions of Unintended Pregnant Women - Counseling and Care Services (Erfahrungen und Lebenslagen ungewollt Schwangerer – Angebote der Beratung und Versorgung)

## References

[1] Janighorban M, Boroumandfar Z, Pourkazemi R, Mostafavi F. Barriers to vulnerable adolescent girls’ access to sexual and reproductive health. BMC Public Health. 2022;22:2212. 10.1186/s12889-022-14687-4. 36447192 10.1186/s12889-022-14687-4 PMC9706928

[2] Morris JL, Rushwan H. Adolescent sexual and reproductive health: the global challenges. Int J Gynaecol Obstet. 2015;131(Suppl 1):S40–2. 10.1016/j.ijgo.2015.02.006. 26433504 10.1016/j.ijgo.2015.02.006

[3] Upadhyay UD, Danza PY, Neilands TB, Gipson JD, Brindis CD, Hindin MJ, et al. Development and validation of the sexual and reproductive empowerment scale for adolescents and young adults. J Adolesc Health. 2021;68:86–94. 10.1016/j.jadohealth.2020.05.031. 32690468 10.1016/j.jadohealth.2020.05.031 PMC7755733

[4] Charmaraman L, McKamey C. Urban early adolescent narratives on sexuality: accidental and intentional influences of family, peers, and the media. Sex Res Soc Policy. 2011;8:253–66. 10.1007/s13178-011-0052-3. 10.1007/s13178-011-0052-3 PMC3440241 22983141

[5] Döring N, Walter R, Scharmanski S. Elterliche Sexualaufklärung und sexuelles Risikoverhalten bei Töchtern und Söhnen: Befunde aus der Repräsentativbefragung „Jugendsexualität“. Bundesgesundheitsbl. 2024;67:14–22. 10.1007/s00103-023-03783-4. 10.1007/s00103-023-03783-4 PMC10776709 37855911

[6] Dehingia N, Barker KM, Raj A. Relationship between adolescent friendship networks and contraceptive use and unintended pregnancies in early adulthood in the united States. Contraception. 2022;110:36–41. 10.1016/j.contraception.2022.02.003. 35202618 10.1016/j.contraception.2022.02.003

[7] Akella D, Jordan M. Impact of social and cultural factors on teen pregnancy. J Health Disparities Res Pract. 2015;8:41–62.

[8] Hoggart L. I’m pregnant … what am i going to do?’ an examination of value judgements and moral frameworks in teenage pregnancy decision making. Health Risk Soc. 2012;14:533–49. 10.1080/13698575.2012.706263.

[9] Pulerwitz J, Blum R, Cislaghi B, Costenbader E, Harper C, Heise L, et al. Proposing a conceptual framework to address social norms that influence adolescent sexual and reproductive health. J Adolesc Health. 2019;64:S7–9. 10.1016/j.jadohealth.2019.01.014. 30914171 10.1016/j.jadohealth.2019.01.014 PMC6426762

[10] Graaf Hde, Schouten F, van Dorsselaer S, Költő A, Ball J, Stevens GWJM, et al. Trends and the gender gap in the reporting of sexual initiation among 15-year-olds: a comparison of 33 European countries. J Sex Res. 2024. 10.1080/00224499.2023.2297906. 38236654 10.1080/00224499.2023.2297906

[11] Graf J, Abele H, Jakubowski P, Teenagerschwangerschaft. gynäkologie + geburtshilfe. 2023;28:22–5. 10.1007/s15013-023-5141-x.

[12] Hall KS, Kusunoki Y, Gatny H, Barber J. Social discrimination, stress, and risk of unintended pregnancy among young women. J Adolesc Health. 2015;56:330–7. 10.1016/j.jadohealth.2014.11.008. 25586228 10.1016/j.jadohealth.2014.11.008 PMC4339533

[13] Duby Z, Bunce B, Fowler C, et al. Who is to blame for the ‘problem’ of teenage pregnancy? Narratives of blame in two South African communities. Reprod Health. 2025;22:18. 10.1186/s12978-025-01958-7. 10.1186/s12978-025-01958-7 PMC11796155 39905545

[14] Smith W, Turan JM, White K, Stringer KL, Helova A, Simpson T, et al. Social norms and stigma regarding unintended pregnancy and pregnancy decisions: a qualitative study of young women in Alabama. Perspect Sex Reprod Health. 2016;48:73–81. 10.1363/48e9016. 27166869 10.1363/48e9016 PMC5022769

[15] Chen X-K, Wen SW, Fleming N, Demissie K, Rhoads GG, Walker M. Teenage pregnancy and adverse birth outcomes: a large population based retrospective cohort study. Int J Epidemiol. 2007;36:368–73. 10.1093/ije/dyl284. 17213208 10.1093/ije/dyl284

[16] Lee D. The early socioeconomic effects of teenage childbearing. DemRes. 2010;23:697–736. 10.4054/DemRes.2010.23.25.

[17] Helfferich C, Hessling A, Klindworth H, Wlosnewski I. Unintended pregnancy in the life-course perspective. Adv Life Course Res. 2014;21:74–86. 10.1016/j.alcr.2014.04.002. 26047543 10.1016/j.alcr.2014.04.002

[18] Sasaki N, Ikeda M, Nishi D. Long-term influence of unintended pregnancy on psychological distress: a large sample retrospective cross-sectional study. Arch Womens Ment Health. 2022;25:1119–27. 10.1007/s00737-022-01273-1. 36306037 10.1007/s00737-022-01273-1

[19] Bermea AM, Toews ML, Wood LG. Students getting pregnant are not gonna go nowhere: manifestations of stigma in adolescent mothers’. Educ Environ Youth Soc. 2018;50:423–36. 10.1177/0044118X16661734.

[20] Machoka BN, Kabiru CW, Ajayi AI. My father insisted that I have the baby but not in his house”: adolescent pregnancy, social exclusion and (dis)empowerment of girls in an urban informal settlement in Kenya. PLoS Glob Public Health. 2024;4:e0003742. 10.1371/journal.pgph.0003742. 39325700 10.1371/journal.pgph.0003742 PMC11426473

[21] Hahn D. Reproduktive gesundheit: Zwischen individuellen Ansprüchen und gesellschaftlichen Realitäten. APuZ. 2024;74:34–40.

[22] Hinz C. 30 Jahre Kairo-Konferenz. APuZ. 2024;74:41–6.

[23] Statistisches Bundesamt (Destatis). Weniger Teenagermütter in Deutschland: 2022 wurden 6 Kinder je 1000 Frauen zwischen 15 und 19 Jahren geboren: Teenagergeburten in den vergangenen zwei Jahrzehnten hierzulande und weltweit stark rückläufig. https://www.destatis.de/DE/Presse/Pressemitteilungen/Zahl-der-Woche/2023/PD23_41_p002.html. (Accessed 28 Dec 2024).

[24] Starrs AM, Ezeh AC, Barker G, Basu A, Bertrand JT, Blum R, et al. Accelerate progress-sexual and reproductive health and rights for all: report of the Guttmacher-Lancet Commission. Lancet. 2018;391:2642–92. 10.1016/S0140-6736(18)30293-9. 29753597 10.1016/S0140-6736(18)30293-9

[25] Jewkes R, Morrell R. Sexuality and the limits of agency among South African teenage women: theorising femininities and their connections to HIV risk practices. Soc Sci Med. 2012;74:1729–37. 10.1016/j.socscimed.2011.05.020. 21696874 10.1016/j.socscimed.2011.05.020 PMC3217103

[26] Miller E, Grace KT, Silverman JG, Decker MR, Alexander KA. Preventing reproductive coercion in adolescence. The Lancet Child & Adolescent Health. 2024;8:91–3. 10.1016/S2352-4642(23)00293-6. 37980919 10.1016/S2352-4642(23)00293-6

[27] Matthiesen S, Block K, Mix S, Schmidt G. Schwangerschaft und Schwangerschaftsabbruch Bei minderjährigen frauen: eine studie Im auftrag des bundesverbands der pro familia, gefördert durch die BZgA. 1st ed. Köln: Bundeszentrale für gesundheitliche Aufklärung (BZgA); 2009.

[28] Eichhorn A. Doing sexual agency: sexuelle Handlungsfähigkeit sexuell missbrauchter Jugendlicher Mädchen in der stationären Jugendhilfe. In: Baar R, Hartmann J, Kampshoff M, Budde J, Messerschmidt A, Maier MS, et al. editors. Geschlechterreflektierte Professionalisierung – Geschlecht und Professionalität in pädagogischen Berufen. Verlag Barbara Budrich; 2019. p. 153–65. 10.3224/jeg.v15i1.09.

[29] Klein A, Zeiske A. Sexualität und Handlungsbefähigung: Ein beitrag Zur Capability-Instrumentenentwicklung. Z Fuer Soziologie Der Erziehung Und Sozialisation. 2009;29:371–86.

[30] Klein V, Becker I, Štulhofer A. Parenting, communication about sexuality, and the development of adolescent womens’ sexual agency: a longitudinal assessment. J Youth Adolesc. 2018;47:1486–98. 10.1007/s10964-018-0873-y. 29881911 10.1007/s10964-018-0873-y

[31] Anda RF, Butchart A, Felitti VJ, Brown DW. Building a framework for global surveillance of the public health implications of adverse childhood experiences. Am J Prev Med. 2010;39:93–8. 10.1016/j.amepre.2010.03.015. 20547282 10.1016/j.amepre.2010.03.015

[32] Felitti VJ, Anda RF, Nordenberg D, Williamson DF, Spitz AM, Edwards V, et al. Relationship of childhood abuse and household dysfunction to many of the leading causes of death in adults. The Adverse Childhood Experiences (ACE) Study. Am J Prev Med. 1998;14:245–58. 10.1016/s0749-3797(98)00017-8. 9635069 10.1016/s0749-3797(98)00017-8

[33] Felitti VJ. The relation between adverse childhood experiences and adult health: turning gold into lead. Perm J. 2002;6:44–7. 10.7812/TPP/02.994. 30313011 10.7812/tpp/02.994 PMC6220625

[34] Felitti VJ. Adverse childhood experiences and adult health. Acad Pediatr. 2009;9:131–2. 10.1016/j.acap.2009.03.001. 19450768 10.1016/j.acap.2009.03.001

[35] Brzank P, Liepe K, Schillmöller Z, Blättner B. Teen dating violence in germany: prevalence, risk factors and impairments. Eur J Public Health. 2014. 10.1093/eurpub/cku166.150.

[36] Born A. Relationship violence and teenage parents. J Infant Child Adolesc Psychother. 2012;11:368–75. 10.1080/15289168.2012.734762.

[37] Barber JS, Kusunoki Y, Gatny H, Melendez R. The relationship context of young pregnancies. Law Inequal. 2017;35:2. https://pmc.ncbi.nlm.nih.gov/articles/PMC5881929/pdf/nihms950903.pdf. PMC5881929 29622858

[38] Parker B, Mcfarlane J, Soeken K, Torres S, Campbell D. Physical and emotional abuse in pregnancy: a comparison of adult and teenage women. Nurs Res. 1993. 10.1097/00006199-199305000-00009. 8506167

[39] Park S, Kim S-H. The power of family and community factors in predicting dating violence: a meta-analysis. Aggress Violent Behav. 2018;40:19–28. 10.1016/j.avb.2018.03.002.

[40] Taussig H, Garrido E. October. Long-Term Impact of a Positive Youth Development Program on Dating Violence Outcomes During the Transition to Adulthood; 2017. https://www.ojp.gov/pdffiles1/nij/grants/251206.pdf (Accessed 28 Dec 2024).

[41] Miller E, Breslau J, Chung W-JJ, Green JG, McLaughlin KA, Kessler RC. Adverse childhood experiences and risk of physical violence in adolescent dating relationships. J Epidemiol Community Health. 2011;65:1006–13. 10.1136/jech.2009.105429. 21321063 10.1136/jech.2009.105429 PMC3686487

[42] Parkes J, Datzberger S, Nagawa R, Musenze JB, Kasidi JR, Bhatia A, et al. Unintended pregnancies in the lives of young people in Luwero, Uganda: a narrative analysis. Cult Health Sex. 2024;26:1201–16. 10.1080/13691058.2024.2305820. 38315580 10.1080/13691058.2024.2305820

[43] Matthiesen S, Schmidt G. Jugendschwangerschaften – kein indikator für sexuelle verwahrlosung. In: Schetsche M, Schmidt R-B, editors. Sexuelle verwahrlosung. Wiesbaden: VS Verlag für Sozialwissenschaften; 2010. pp. 119–43. 10.1007/978-3-531-92477-9_6.

[44] Ajayi AI, Odunga SA, Oduor C, Ouedraogo R, Ushie BA, Wado YD. I was tricked”: understanding reasons for unintended pregnancy among sexually active adolescent girls. Reprod Health. 2021;18:19. 10.1186/s12978-021-01078-y. 33482843 10.1186/s12978-021-01078-y PMC7821647

[45] Bohnsack R, Pfaff N, Weller W, editors. Qualitative analysis and documentary method in international educational research. Opladen; Farmington Hills, MI: Verlag Barbara Budrich; 2010. https://www.pedocs.de/volltexte/2020/18255/pdf/Bohnsack_Pfaff_Weller_2010_Qualitative_Analysis_and_Documentary_Method.pdf.

[46] Verbundprojekt ELSA. Abschlussbericht der Studie: Erfahrungen und Lebenslagen ungewollt Schwangerer. Angebote der Beratung und Versorgung (ELSA). 2024. https://www.bundesgesundheitsministerium.de/fileadmin/Dateien/5_Publikationen/Gesundheit/Abschlussberichte/ELSA_Abschlussbericht.pdf.

[47] Schütze F. Biographieforschung und narratives interview. Neue Praxis. 1983;13:283–93.

[48] Nohl A-M. Interview und dokumentarische methode. Wiesbaden: Springer Fachmedien Wiesbaden; 2017.

[49] Bohnsack R, Nentwig-Gesemann I, editors. Dokumentarische evaluationsforschung: theoretische grundlagen und beispiele Aus der praxis. Opladen: Budrich; 2010.

[50] Przyborski A. Qualitative sozialforschung: Ein arbeitsbuch. 4th ed. Berlin/München/Boston: Walter de Gruyter GmbH; 2014.

[51] Stützel K. Jugendarbeit Im kontext von Jugendlichen Mit rechten orientierungen. Wiesbaden: Springer Fachmedien Wiesbaden; 2019.

[52] Bohnsack R, Rekonstruktive, Sozialforschung. Einführung in qualitative methoden. 10th ed. Opladen, Toronto: Verlag Barbara Budrich; 2021. http://www.blickinsbuch.de/item/169daf895baeb9e5aff2464436ba288d.

[53] Liu Y, Huang X. Effects of basic psychological needs on resilience: A human agency model. Front Psychol. 2021;12:700035. 10.3389/fpsyg.2021.700035. 34531790 10.3389/fpsyg.2021.700035 PMC8438124

[54] Klann EM, Wong YJ. A pregnancy Decision-Making model: Psychological, Relational, and cultural factors affecting unintended pregnancy. Psychol Women Q. 2020;44:170–86. 10.1177/0361684320904321.

[55] Klann EM, Wong YJ, Pregnancy Decision Making for Women With Mental Health Conditions. The roles of Distress, Lay Beliefs, and Self-Efficacy. Psychol Women Q. 2023;47:547–61. 10.1177/03616843231191505.

[56] Bannwart J. Die ambivalenz der „Abtreibung: eine analyse der Kontroverse Um Den „Schwangerschaftsabbruch Anhand des konzeptes Zu abweichendem verhalten von Howard S. Becker. Sozialpolitik Ch. 2018. 10.18753/2297-8224-108.

[57] Becker HS, Außenseiter. Zur soziologie abweichenden verhaltens. Wiesbaden: Springer Fachmedien Wiesbaden; 2014.

[58] Wörner L, Stetter-Karp I. Entkriminalisierung des schwangerschaftsabbruchs? Zwei perspektiven. APuZ. 2024;74:23–33.

[59] Bundesministerium der Justiz. German Criminal Code (StGB): § 218 Section Abortion. 1993. https://www.gesetze-im-internet.de/englisch_stgb/englisch_stgb.html#p1254 (Accessed 28 Dec 2024).

[60] Herrenkohl TI, Fedina L, Roberto KA, Raquet KL, Hu RX, Rousson AN, et al. Child maltreatment, youth violence, intimate partner violence, and elder mistreatment: a review and theoretical analysis of research on violence across the life course. Trauma Violence Abuse. 2022;23:314–28. 10.1177/1524838020939119. 32723166 10.1177/1524838020939119 PMC10202370

[61] Brzank P. Wege Aus der Partnergewalt. Wiesbaden: VS Verlag für Sozialwissenschaften; 2012.

[62] Jung A, Korn A, Winter K, Brzank PJ. Vulnerabilitäten und handlungsmacht von ungewollt Schwangeren Mit partnergewalterfahrungen: die Komplexität der gleichzeitigkeit. Psychol Gesellschaftskrit. 2023;47:357–81.

[63] Ragavan MI, Culyba AJ, Shaw D, Miller E. Social support, exposure to parental intimate partner violence, and relationship abuse among marginalized youth. J Adolesc Health. 2020;67:127–30. 10.1016/j.jadohealth.2020.01.020. 32165077 10.1016/j.jadohealth.2020.01.020 PMC8084302

[64] Haj-Yahia MM, Sokar S, Hassan-Abbas N, Malka M. The relationship between exposure to family violence in childhood and post-traumatic stress symptoms in young adulthood: the mediating role of social support. Child Abuse Negl. 2019;92:126–38. 10.1016/j.chiabu.2019.03.023. 30974256 10.1016/j.chiabu.2019.03.023

[65] Wood SK, Ford K, Madden HCE, Sharp CA, Hughes KE, Bellis MA. Adverse childhood experiences and their relationship with poor sexual health outcomes: results from four cross-sectional surveys. Int J Environ Res Public Health. 2022. 10.3390/ijerph19148869. 35886718 10.3390/ijerph19148869 PMC9316235

[66] Hughes K, Bellis MA, Hardcastle KA, Sethi D, Butchart A, Mikton C, et al. The effect of multiple adverse childhood experiences on health: a systematic review and meta-analysis. Lancet Public Health. 2017;2:e356–66. 10.1016/S2468-2667(17)30118-4. 29253477 10.1016/S2468-2667(17)30118-4

[67] Bellis MA, Hughes K, Leckenby N, Perkins C, Lowey H. National household survey of adverse childhood experiences and their relationship with resilience to health-harming behaviors in England. BMC Med. 2014;12:72. 10.1186/1741-7015-12-72. 24886026 10.1186/1741-7015-12-72 PMC4234527

[68] World Health Organization (WHO). WHO multi-country study on women’s health and domestic violence against women: initial results on prevalence, health outcomes and women’s responses. Geneva, Switzerland: World Health Organization; 2005.

[69] Bordogna AL, Coyle AC, Nallamothu R, Manko AL, Yen RW. Comprehensive sexuality education to reduce pregnancy and stis in adolescents in the united states: a systematic review and meta-analysis. Am J Sex Educ. 2023;18:39–83. 10.1080/15546128.2022.2080140.

[70] Pires R, Araújo-Pedrosa A, Pereira J, Canavarro MC. How can unintended pregnancies be prevented among adolescents who engaged in sexual intercourse at earlier ages? The role of female education and partner age difference. Int J Environ Res Public Health. 2021. 10.3390/ijerph182010631. 34682377 10.3390/ijerph182010631 PMC8535923

[71] Oringanje C, Meremikwu MM, Eko H, Esu E, Meremikwu A, Ehiri JE. Interventions for preventing unintended pregnancies among adolescents. Cochrane Database Syst Rev. 2016;(2):CD005215. 10.1002/14651858.cd005215.pub3. 10.1002/14651858.CD005215.pub3 PMC8730506 26839116

[72] Scharmanski S, Hessling A. Sexualaufklärung Junger menschen in Deutschland. Ergebnisse der repräsentativen Wiederholungsbefragung „Jugendsexualität. J Health Monit. 2022;23–41. 10.25646/9874.

[73] Mohamed S, Chipeta MG, Kamninga T, Nthakomwa L, Chifungo C, Mzembe T, et al. Interventions to prevent unintended pregnancies among adolescents: a rapid overview of systematic reviews. Syst Rev. 2023;12:198. 10.1186/s13643-023-02361-8. 37858208 10.1186/s13643-023-02361-8 PMC10585784

[74] Alzate MM, Dongarwar D, Matas JL, Salihu HM. The effect of sexual literacy on adolescent pregnancy in Colombia. J Pediatr Adolesc Gynecol. 2020;33:72–82. 10.1016/j.jpag.2019.09.005. 31561033 10.1016/j.jpag.2019.09.005

[75] Obelenienė B. From birth control to self-awareness and free decision making. A model for the evaluation of comprehensive sexuality education from the perspective of women’s health and free informed choice. The Pontifical University of John Paul II in Krakow. 2022. 10.15633/9788363241513.

[76] Rowlands A, Juergensen EC, Prescivalli AP, Salvante KG, Nepomnaschy PA. Social and biological transgenerational underpinnings of adolescent pregnancy. Int J Environ Res Public Health. 2021. 10.3390/ijerph182212152. 34831907 10.3390/ijerph182212152 PMC8620033

[77] Mersky JP, Topitzes J, Britz L. Promoting evidence-based, trauma-informed social work practice. J Social Work Educ. 2019;55:645–57. 10.1080/10437797.2019.1627261.

[78] Levenson J. Trauma-informed social work practice. Soc Work. 2017;62:105–13. 10.1093/sw/swx001. 28339563 10.1093/sw/swx001

[79] Abraham A. He did exactly what they probably did to him: A qualitative study on transgenerational effects of foster Care-Related experiences of violence. In: Sinha R, Basu P, editors. Family and gendered violence and conflict. Cham: Springer Nature Switzerland; 2024. pp. 417–34. 10.1007/978-3-031-60383-9_23.

